# Cytotoxicity Evaluation of Novel bis(2-aminoethyl)amine Derivatives

**DOI:** 10.3390/molecules25122816

**Published:** 2020-06-18

**Authors:** Daniel Szulczyk, Anna Bielenica, Piotr Roszkowski, Michał A. Dobrowolski, Wioletta Olejarz, Mariola Napiórkowska, Marta Struga

**Affiliations:** 1 Chair and Department of Biochemistry, Medical University, 02–097 Warszawa, Poland; abielenica@wum.edu.pl (A.B.); mariola.napiorkowska@wum.edu.pl (M.N.); marta.struga@wum.edu.pl (M.S.); 2 Faculty of Chemistry, University of Warsaw, Pasteura 1, 02-093 Warsaw, Poland; roszkowski@chem.uw.edu.pl (P.R.); miked@chem.uw.edu.pl (M.A.D.); 3 Department of Biochemistry and Pharmacogenomics, Faculty of Pharmacy, Medical University of Warsaw, 02-097 Warszawa, Poland; wioletta.olejarz@wum.edu.pl; 4 Laboratory of Centre for Preclinical Research, Medical University of Warsaw, Banacha 1B, 02-097 Warsaw, Poland

**Keywords:** bis(2-aminoethyl)amine, thiourea, tetrazole, cytotoxicity, crystal structure

## Abstract

Seven novel derivatives of bis(2-aminoethyl)amine were synthesized. For compounds **1** and **7** single crystals were isolated and X-ray diffraction experiments were done. Lipophilicity and drug likeness were calculated in the initial stage of research. All compounds were screened for their in vitro cytotoxic activity against a panel of human cancer cell lines, which is contrary to normal (HaCaT) cell lines, by using the MTT method. Studies were followed by lactate dehydrogenase assay, apoptotic activity, and interleukin-6 assay. Within the studied group, compound **6** showed the most promising results in all biological studies. The strongest influence in A549 cells was denoted for derivative **4**, which inhibited interleukin release almost tenfold, as compared to the control.

## 1. Introduction

Derivatives of bis(2-aminoethyl)amine and (thio)urea are poorly investigated for their biological activities. Limited papers related to such compounds can be found. Few similar structures were found to be suitable spacers for sulphate and phosphate receptors, transmembrane transporters for bicarbonate, and synthetic receptors [1,2,3].

**Figure 1:**
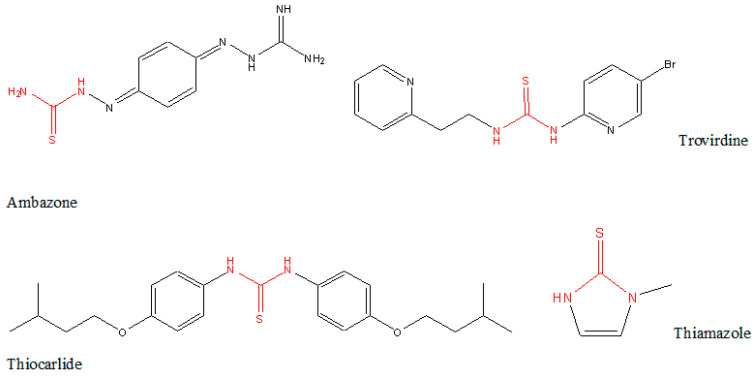
Structure of antiseptic Ambazone, HIV-1 RT inhibitor Trovirdine, antimycobacterial Thiocarlide, used to treat hyperthyroidism Thiamazole.

Thiourea moiety is commonly used in designing synthesis schemes and in the research for new compounds possessing biological activity. Some of the outcomes are commercialized and commonly used as medicines. Few examples of structures with diverse activity are given below (Figure 1) [4].

Linear and cyclic thiourea derivatives have been intensively studied. From the synthetic point of view, this motif was used to obtain mono-, di-, and cyclic substituted compounds [5,6,7,8,9]. Most of them were built on a heterocyclic scaffold, which is sometimes a complex one [10,11,12,13]. Most of these compounds showed significant biological activity. In some of reported studies, researchers were using thiourea derivatives as substrates to replace thiourea motif by heterocyclic arrangement [14,15,16,17]. Wide spectra of biological activities are reported continuously for thiourea-based compounds [18,19,20,21]. The number of derivatives were design and studied for their anti-cancer properties. In published papers, future anti-cancer candidates were screened in tests in vitro using tissue cells.

Cytotoxicity is one of the most important indicators for biological evaluation in vitro studies. Chemicals such as medicines have different cytotoxicity mechanisms such as destruction of cell membranes, prevention of protein synthesis, irreversible binding to receptors, etc. In order to determine the cell death caused by these damages, there is a need for reliable and reproducible short-term cytotoxicity and cell viability assays. Both assays are based on various cell functions. A broad spectrum of cytotoxicity assays is currently used in the fields of toxicology and pharmacology [22]. These tests are commonly used as screening tests for compounds targeting cancer cells. The primary idea is to observe the normal and mutated cells growth, reproduction, and morphological effects. Cytotoxicity is preferred as a pilot project test and an important indicator for toxicity evaluation.

Prompted by preliminary test results and years of thiourea derivatives synthesis, we have decided to conduct a study on a group of new promising compounds. Our main goal was to determine if bis(2-aminoethyl)amine thiourea derivatives could be recognized as an interesting class of anti-cancer drugs based on cytotoxicity assay results.

## 2. Results and Discussion

### 2.1. Chemistry

**Scheme 1:**
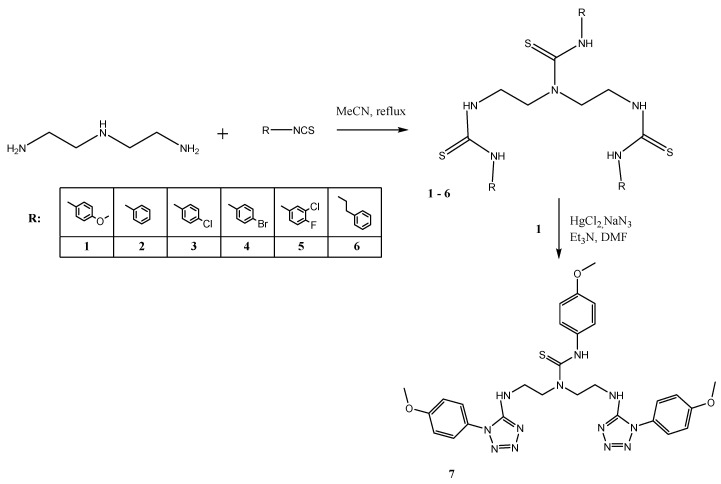
Six 3-substitued-1,1-bis[2-(3-substituted-thioureido)ethyl]thioureas and 3-(4-methoxyphenyl)-1,1-bis(2-{[1-(4-methoxyphenyl)-1H-tetrazol-5-yl]amino}ethyl)thiourea synthetic path.

We have planned to obtain a seven bis(2-aminoethyl)amine thiourea derivatives. A synthetic route is depicted below (Scheme 1).

Bis(2-aminoethyl)-amine was heated with suitable isothiocyanates to give six thiourea derivatives. The reaction protocol is well known. Therefore, there is no need for further discourse [17]. The seventh compound was obtained in a desulfurization/cyclization reaction using the mercuric chloride and sodium azide. The mechanism of this type of reaction is established (Scheme 1). Therefore, our goal was to find suitable reaction conditions [17]. It was confirmed that no reflux is necessary. Furthermore, catalytic amounts of triethylamine (1–3 drops) are optional. Additional information can be found in the experimental part.

For synthesized compounds, lipophilicity was calculated using Moriguchi LOGP computational methodology [23,24]. Furthermore, drug likeness was assessed by the Lipinski rule of five [25]. Calculation were done using SwissADME tool and summarized below (Table 1) [26].

It was decided to design only seven compounds because of large molar weight, which was represented by a violation of Lipinski rule of five in each structure. The octanol-water partition coefficient is expressed by Log P, which ranged from 2.17 to 5.64. Thus, synthesized derivatives should be considered as possessing diverse lipophilicity. Four of the compounds **3, 4, 5, 7** violated two of the Lipinski rules. Derivative **7** was synthesized using **1** as starting material for two main reasons. First, transition from thiourea to tetrazole derivative practically does not change molar weight, but has impact on lipophilicity. Second, our previous studies showed that this transition is extremely profitable in terms of designed pharmacological action [16,17]. It was decided to synthesize only one tetrazole compound at the first stage of studies to compare cytotoxicity results between thiourea and tetrazole derivative.

### 2.2. X-Ray Studies

For compounds **1** and **7,** single crystals were isolated and X-ray diffraction experiments were done. Crystal data and structural refinement for analyzed compounds are summarized in Table 2.

**Figure 2:**
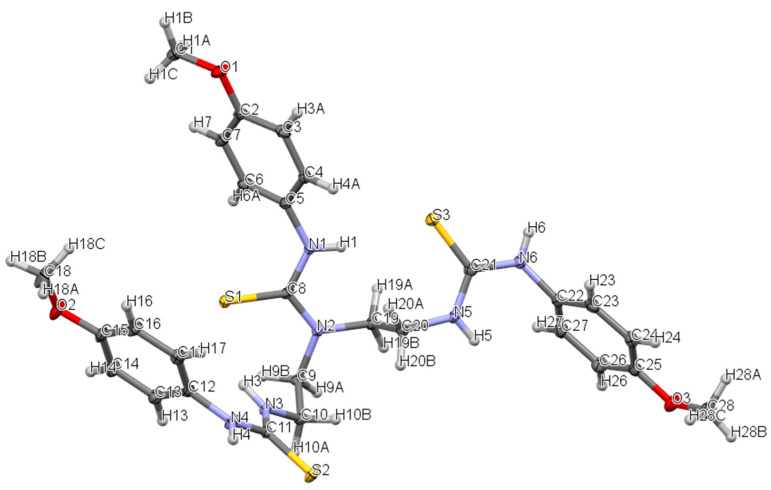
The molecular structure of 3-(4-methoxyphenyl)-1,1-bis{2-[3-(4-methoxyphenyl)thioureido]ethyl}thiourea (**1**) showing atom numbering and displacement ellipsoids at the 50% probability level.

**Figure 3:**
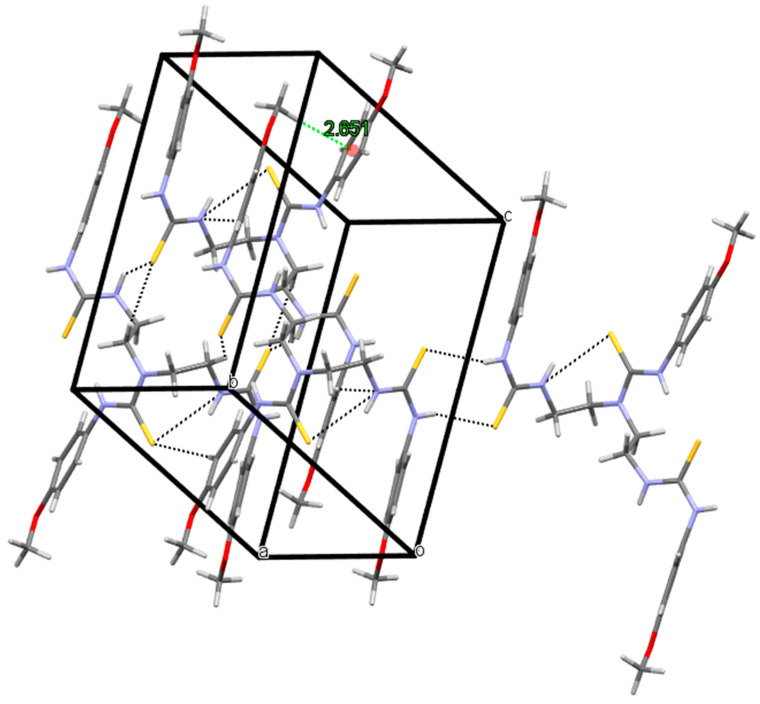
The packing and selected interactions in the crystal lattice of 3-(4-methoxyphenyl)-1,1-bis{2-[3-(4-methoxyphenyl)thioureido]ethyl}thiourea (**1**).

3-(4-methoxyphenyl)-1,1-bis{2-[3-(4-methoxyphenyl)thioureido]ethyl}thiourea (**1**) crystallizes in a triclinic system, space group P-1, with one molecule in an asymmetric part of the unit cell (Figure 2). There are intramolecular and intermolecular N-H…S hydrogen bonds in the crystal lattice: N3-H3…S1 with distance 2.550 Å, N4-H4…S2 with distance 2.577 Å (adjacent molecules form dimers) and N1-H1…S3 with distance 2.593 Å. Additionally, the structure is stabilized by C-H…π interactions (the distance between the hydrogen atom and the centroid of the phenyl ring is 2.651 Å, see Figure 3) and weak C-H…S, C-H…N contacts (distance range 2.651–3.004 Å). The crystal packing and short interactions are shown in Figure 3.

**Figure 4:**
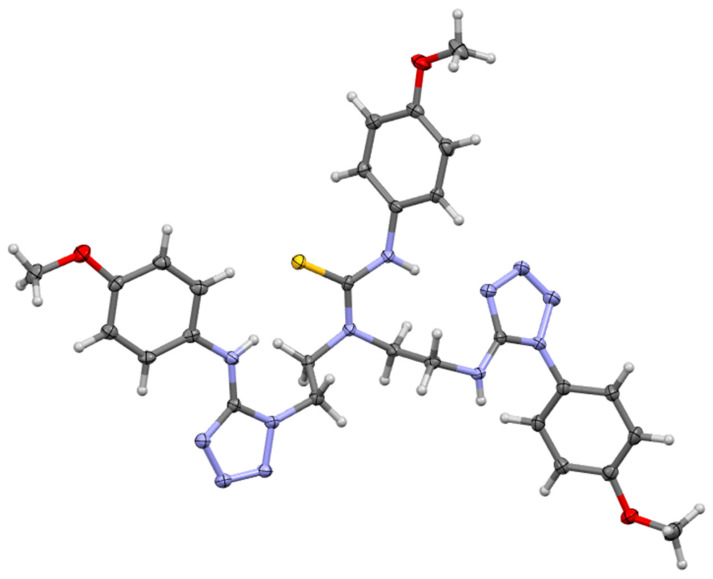
The molecular structure of 3-(4-methoxyphenyl)-1,1-bis(2-{[1-(4-methoxyphenyl)-1H-tetrazol-5-yl]amino}ethyl)thiourea (**7**) showing atom numbering and displacement ellipsoids at the 50% probability level.

**Figure 5:**
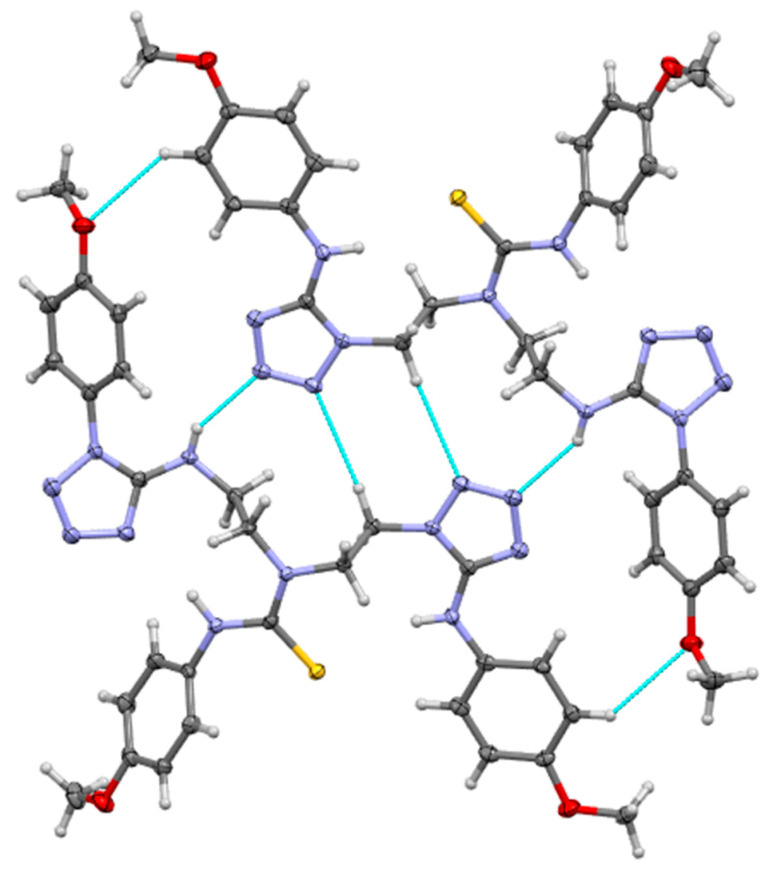
Selected interactions in the crystal lattice of 3-(4-methoxyphenyl)-1,1-bis(2-{[1-(4-methoxyphenyl)-1H-tetrazol-5-yl]amino}ethyl)thiourea (**7**).

3-(4-methoxyphenyl)-1,1-bis(2-{[1-(4-methoxyphenyl)-1H-tetrazol-5-yl]amino}ethyl)thiourea (**7**) also crystallizes in a triclinic system, space group P-1, with one molecule in an asymmetric part of the unit cell (Figure 4). There are strong intermolecular N-H…N hydrogen bonds in the crystal lattice with distance 2.221 Å forcing adjacent molecules to form dimers (see Figure 5). Additionally, the structure is stabilized by C-H…O interactions with distances equal to 2.709 Å and weak N-H…N contacts (2.798 Å). The hydrogen bonds and short interactions are shown in Figure 5.

### 2.3. Biological Studies

#### 2.3.1. Anti-Cancer Activity

All derivatives of bis(2-aminoethyl)amine were screened for their in vitro cytotoxic activity against a panel of human cancer cell lines, namely colorectal adenocarcinoma (CaCo-2), epithelial lung carcinoma (A549), and melanoma (HTB-140), which is contrary to normal (HaCaT) cell lines, by using the MTT method.

The tested compounds exhibited a moderate antiproliferative potency (Table 3). The highest growth-inhibitory activity on all cancer cell lines was denoted for phenethyl derivative **6** (IC_50_ from 13.95 ± 2.5 to 15.74 ± 1.7 µM).

A similar level of an anti-proliferative action was observed for thioureas with electron-withdrawing substituents on the phenyl ring such as 4-bromo-(**4)** (14.74 ± 1.5 µM), 3-chloro-4-fluoro- (**5)** (17.87 ± 2.8 µM), and, to a lesser extent, 4-chlorophenylthiourea **3** (22.63 ± 2.0 to 27.70 ± 3.7 µM). The selectivity index (SI) for evaluated compounds was higher than 1 (1.02–1.35). Unsubstituted phenylthiourea **2** and derivatives containing electron-donating 4-methoxy group on the benzene ring (**1**, **7**) remained inactive toward chosen cancer and normal cell lines. 

The HTB-140 cell line was the most sensitive to the presence of tested derivatives. The selectivity factors for this line were also more explicit (SI ≥ 1.16). SI of reference cytostatics toward evaluated cells are also low (1.46–2.51).

Moreover, a link between anti-cancer potency and calculated logP values of new compounds was found. The most cytotoxic thioureas (**3–6**) are characterized by the highest octanol-water partition coefficients (3.51–4.83). However, in contrast to other active derivatives, the most promising phenethylthiourea **6**, with logP 3.51, complied with the rule of five. Substances **1**, **2**, and **7** described by indexes ranged from 2.17 to 3.47, which remained biologically inactive. 

The strong dependence between cytotoxic activity and the presence of electronegative elements on the phenyl moiety or an alkyl linker at the thiourea part, was proven by our team previously [5,6,7,8,9,12,13,14,15,16,17].

#### 2.3.2. Lactate Dehydrogenase Assay

Lactate dehydrogenase assay (LDH) as a marker of cell death was evaluated for synthesized ethylthioureas **1–7**. The compounds were tested at concentrations of 10, 50, and 100 µM. At the lowest dose, no activity was observed.

**Figure 6:**
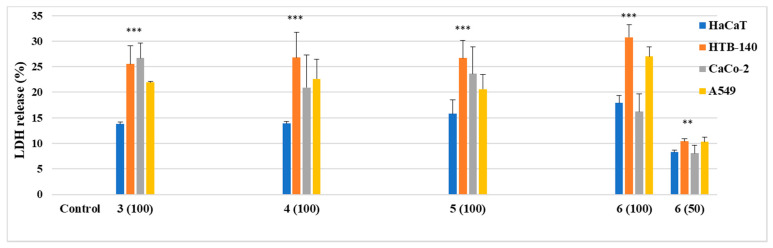
LDH release as a marker of a cell death in the HaCaT, HTB-140, CaCo-2, A549 cells, treated for 72 h with various concentrations of derivatives **3–6** (100 µM for compounds **3–5**, 100 and 50 µM for **6**). Data are expressed as the mean ± SD from three independent experiments, performed in triplicate. *** *p* ≤ 0.001, ** *p* ≤ 0.01, as compared to the control.

As shown in Figure 6, the LDH test highlighted relevantly lower toxicity of the new derivatives (at 100 µM) against HaCaT cells, as compared to cancer cell lines.

The LDH release from normal keratinocytes ranged from 13.8% (compound **3**) to 17.9% (compound **6**). When a dose of 50 µM of the phenethyl derivative **6** was applied, it equaled 8.3%. Synthesized substances **3–6**, studied at 100 µM, expressed moderate cytotoxic potency against tumor cells. However, the effect was stronger in the melanoma cells (HTB-140) when compared to colorectal adenocarcinoma (CaCo-2) and lung carcinoma cell lines (A549). The LDH secretion from HTB-140 cells varied from 25.5% to 30.8%, and the derivative **6** showed the highest response. In addition, the compounds were less cytotoxic for A549 and CaCo-2 cell lines, achieving from 16.2% to 27.0% LDH release. 

The thiourea **6** exerted more evident cytotoxic ability against two tested cell lines: HTB-140 and A549, whereas the halogen derivative **3** was found to be the most effective in the LDH releasing from CaCo-2 cells. The secretion of dehydrogenase in the presence of the compound **6**, applied at 50 µM, was similar for all chosen cell lines, which are both normal (8.3%) and pathological (8.09–10.4%). 

The data achieved by the LDH assay are in agreement with the results obtained by the MTT method.

#### 2.3.3. Apoptotic Activity 

**Figure 7:**
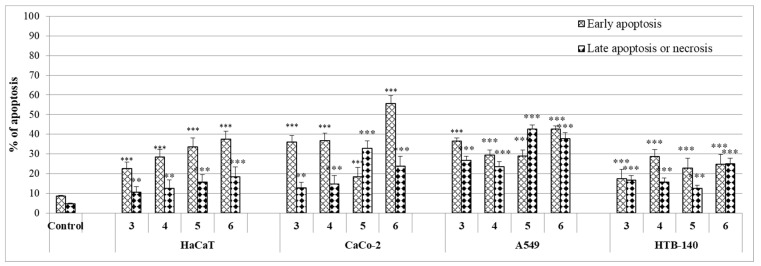
The effect of derivatives **3**–**6** on early and late apoptosis, or necrosis in HaCaT, CaCo-2, A549, and HTB-140 cells. Cells were incubated for 72 h with tested compounds and used in their IC_50_ concentrations. Then cells were harvested, stained with Annexin V-FITC and 7-AAD, and analyzed using flow cytometry. Data are expressed as the percentage of cells at an early stage of apoptosis or late stage of apoptosis or necrosis. Data are given as means ± SD, *** *p* ≤ 0.001, ** *p* ≤ 0.01, as compared to the control.

To establish in vitro, the anti-proliferative mechanism of activity, the influence of bis(2-aminoethyl)amine derivatives on early and late apoptosis was performed, using flow cytometry analysis (Figure 7).

**Figure 8:**
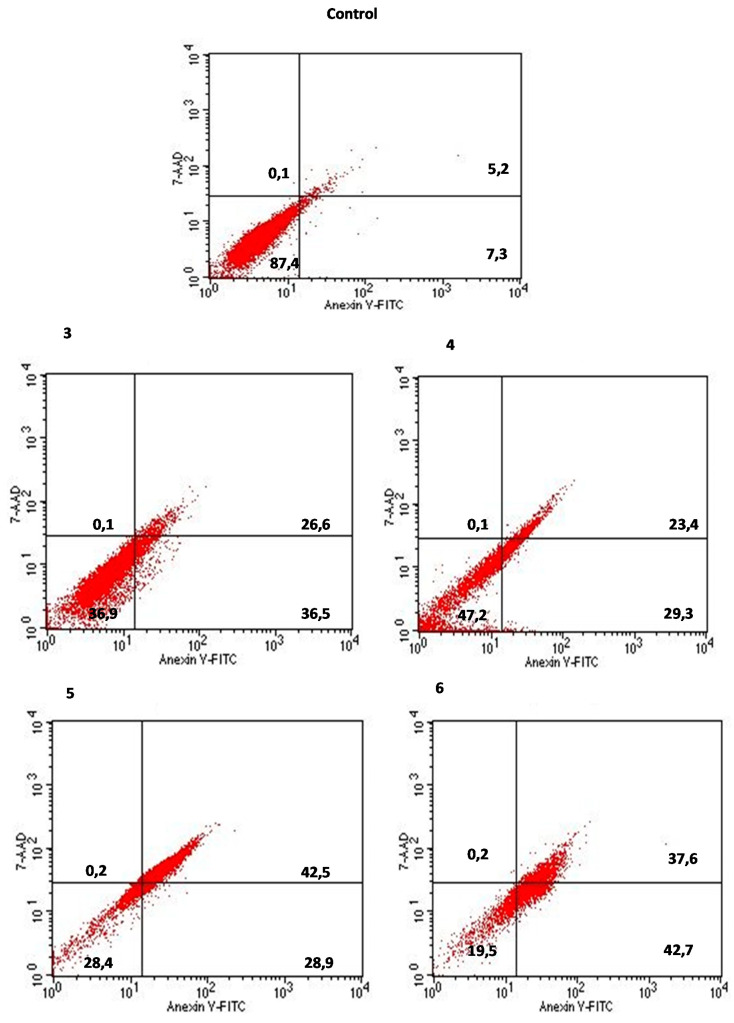
The effect of compounds **3**–**6** on early and late apoptosis or necrosis in A549 cells, detected with Annexin V-FITC/7-AAD by flow cytometry. The lower right quadrant shows early apoptotic cells (Annexin V-FITC positive and 7-AAD negative staining). The upper right and upper left quadrants represent the late stage of apoptotic or necrotic cells (Annexin V-FITC positive, 7-AAD positive, Annexin V-FITC negative and 7-AAD positive staining, respectively). The lower left quadrant represents unstained A549 cells (Annexin V-FITC negative, 7-AAD negative).

The treatment of lung carcinoma (A547) cells with compounds **3**–**6** showed noticeably higher percentage of cells in early apoptosis (28.9–42.7%) as compared to the control (7.3%) (Figure 8).

Thiourea compounds **5** and **6** induced the late apoptosis in these cells in 42.7% and 37.8%, respectively. The late apoptosis inducing properties of derivatives **3** and **4** were weaker (23.5% and 26.7%, respectively). In general, the alkylphenyl derivative **6** was the most potent, effecting not only on A549 cells apoptosis, but also considerably (in 55.7%) inducing the early apoptosis in CaCo-2 cell lines.

Halogen-containing compounds **3** and **4** indicated marked pro-apoptic inducing properties in CaCo-2 cell lines. The early-apoptotic activity of these derivatives varied from 35.9% to 36.7%. In contrast, the 3-chloro-4-fluorophenylthiourea **5** increased mainly late apoptosis of these cells (32.8%). Apoptosis in HTB-140 cells was not spectacular. However, bromophenyl derivative **4** affected the level of early apoptosis in 28.6% (see Supplementary Material). 

The obtained results comply with IC_50_ data assigned to CaCo-2 and A549 cell lines by using the MTT method (Table 3).

#### 2.3.4. Interleukin-6 Assay

The mechanism of cytotoxic action of bis(2-aminoethyl)-amine derivatives can be associated with reducing interleukin-6 (IL-6) levels, which include the strong pro-inflammatory particles. 

**Figure 9:**
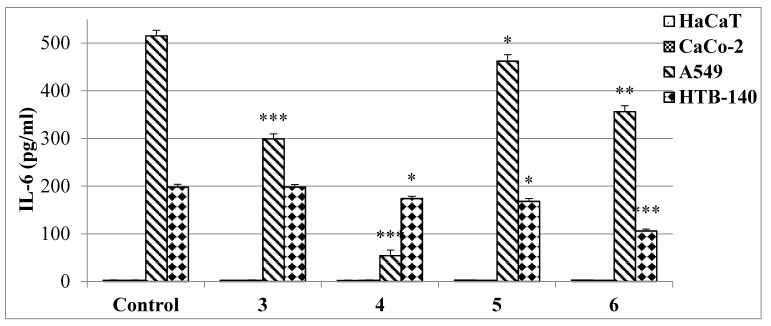
Effects of compounds **3**–**6** on IL-6 levels, measured by the ELISA test. Data are expressed as the mean ± SD from three independent experiments, performed in triplicate. *** *p* ≤ 0.001, ** *p* ≤ 0.01, * *p* ≤ 0.05, as compared to the control.

All investigated human cell lines were treated with IC_50_ doses of the most potent derivatives **3–6** (Figure 9).

The strongest influence in A549 cells was denoted for 4-bromophenyl derivative **4**, which inhibited interleukin release almost tenfold, as compared to the control. The treatment of these cells with the 4-chlorophenylthiourea **3** was less effective since that compound decreased the IL-6 concentration only 1.7 times. On the other hand, the phenethyl derivative **6** inhibited IL-6 secretion in HTB-140 cells two-fold.

To conclude, the highest anti-inflammatory effect of studied compounds was observed in A549 and HTB-140 cells. The CaCo-2 cell line was insensitive for the inhibition of IL-6 release, evoked by the presence of tested bis(2-aminoethyl)amine derivatives.

## 3. Conclusions

Designed bis(2-aminoethyl)amine derivatives **1–7** were obtained and transferred to further biological studies supported by appropriate lipophilicity and drug-likeness calculations. Compounds **1, 2,** and **7** were found less active in conducted tests. Based on results and our previous studies, we can conclude that phenyl-electron withdrawing substituents are fundamental to achieve the desired level of activity. However, single crystals isolated for **1** and **7** gained valuable physicochemical data related to obtained structures.

As a result of conducted biological studies, it was found that the highest growth-inhibitory activity on all cancer cell lines was denoted for phenethyl compound **6** (IC_50_ from 13.95 ± 2.5 to 15.74 ± 1.7 µM). The LDH secretion from HTB-140 cells varied from 25.5% to 30.8%, and the **6** showed the highest response. In general, the phenethyl derivative **6** was the most potent, effecting not only on A549 cells apoptosis, but also considerably (in 55.7%) inducing the early apoptosis in CaCo-2 cell lines. However, the strongest influence in A549 cells was denoted for 4-bromophenyl compound **4**, which inhibited interleukin release almost ten-fold, as compared to the control.

Summarizing, bis(2-aminoethyl)amine derivatives should be considered as promising in the field of anti-cancer action. Further research will be performed on a broader group of compounds supported by structure-activity relationship (SAR) studies.

## 4. Materials and Methods

### 4.1. Apparatus, Materials, and Analysis

The reagents were supplied from Alfa Aesar (Haverhill, MA, USA) or Sigma Aldrich (Saint Louis, MO, USA). Organic solvents (acetonitrile, DMF, chloroform, and methanol) were supplied from POCh (Polskie Odczynniki Chemiczne, Gliwice, Poland). All chemicals were of analytical grade. Before use, dried DMF and acetonitrile were kept in crown cap bottles over anhydrous phosphorus pentoxide (Carl Roth, Karlsruhe, Germany).

The NMR spectra were recorded on a Bruker AVANCE DMX400 (Bruker, Billerica, MA, USA) spectrometer, operating at 300 MHz (^1^H NMR) and 75 MHz (^13^C NMR). The chemical shift values are expressed in ppm relative to TMS as an internal standard. Mass spectral ESI measurements were carried out on Waters ZQ Micro-mass instruments (Waters, Milford, MA, USA) with a quadrupol mass analyzer. The spectra were performed in the positive ion mode at a de-clustering potential of 40–60 V. The sample was previously separated on a UPLC column (C18) using the UPLC ACQUITY^TM^ system by Waters connected with a DPA detector. Flash chromatography was performed on Merck silica gel 60 (200–400 mesh) using chloroform/methanol (19:1 vol) mixture as eluent. Analytical TLC was carried out on silica gel F_254_ (Merck) plates (0.25 mm thickness). 

X-ray diffraction experiments were performed at 100(2) K and 130(2) for (**1**) and (**7**), respectively, on a Bruker D8 Venture Photon100 diffractometer (Bruker, Billerica, MA, USA) with a TRIUMPH monochromator and MoK_α_ fine-focus sealed tube (λ = 0.71073 Å).

The crystals were positioned 33 and 30 mm from the PHOTON II detector with CPAD technology for (**1**) and (**7**), respectively. A total of 681 and 579 frames were measured with exposure times of 3.78 and 3.22 h for (**1**) and (**7**). Data were collected using the APEX2 program [27], integrated with the Bruker SAINT software package [28] and corrected for absorption effects using the multi-scan method (SADABS) [29]. The structures were solved and refined using the SHELX software package [30] and Olex2 program [31] using Least Squares minimization. The atomic scattering factors taken from the International Tables [32]. The figures were generated with Mercury (ver. 4.2.0) [33].

CCDC 2003733-2003734 contains the supplementary crystallographic data for this paper. These data can be obtained free of charge via http://www.ccdc.cam.ac.uk/conts/retrieving.html.

### 4.2. Derivatives of 3-Substitued-1,1-bis(2-(3-substituted-thioureido)ethyl)thioureas

#### General Procedure

A solution of bis(2-aminoethyl)-amine (0.07 mol) in acetonitrile (25 mL) was treated with appropriate isothiocyanate (0.075 mol) and the mixture was refluxed for 8 h. Then solvent was removed on a rotary evaporator. The residue was purified by column chromatography on silica gel (chloroform: methanol, 9.5:0.5 vol.).

3-(4-methoxyphenyl)-1,1-bis{2-[3-(4-methoxyphenyl)-thioureido]ethyl}thiourea (**1**)

Yield 89%. ^1^H NMR (300 MHz, DMSO-d_6_): δ 3.73–3.74 (m, 13H, CH_2_NH and OCH_3_), 3.85–3.94 (m, 4H, NCH_2_), 6.86–6.92 (m, 6H, ArH *o*-NH), 7.18–7.22 (m, 6H, ArH *o*-OCH_3_), 7.68 (bs, 2H, NHCH_2_), 9.26 (s, 1H, NHAr), 9.53 (bs, 2H, NHAr); ^13^C NMR (75 MHz, DMSO-d_6_): δ 41.5 (2C, CH_2_), 49.5 (2C, CH_2_), 55.2 (OCH_3_), 55.3 (2C, OCH_3_), 113.2 (2C, ArC *o*-OCH_3_), 114.1 (4C, ArC *o*-OCH_3_), 126.4 (4C, ArC *o*-NH), 127.9 (2C, ArC *o*-NH), 131.1 (ArC *p*-OCH_3_), 133.2 (2C, ArC *p*-OCH_3_), 156.8 (ArC *p*-NH), 156.9 (2C, ArC *p*-NH), 180.9 (2C, C=S), 181.1 (C=S).

3-phenyl-1,1-bis[2-(3-phenylthioureido)ethyl]thiourea (**2**)

Yield 78%. ^1^H NMR (300 MHz, DMSO-d_6_): δ 3.76–3.80 (m, 4H, CH_2_NH), 3.94–3.97 (m, 4H, NCH_2_), 7.11–7.19 (m, 3H, ArH *p*-NH), 7.28–7.38 (m, 12H, ArH), 7.91 (bs, 2H, NHCH_2_), 9.34 (s, 1H, NHAr), 9.72 (bs, 2H, NHAr); ^13^C NMR (75 MHz, DMSO-d_6_): δ 41.3 (2C, CH_2_), 49.5 (2C, CH_2_), 123.7 (ArC *p*-NH), 124.7 (2C, ArC *p*-NH), 125.0 (2C, ArC *o*-NH), 126.3 (4C, ArC *o*-NH), 128.0 (ArC *p*-NH), 128.8 (2C, *p*-NH), 138.7 (ArC, NHAr), 140.4 (2C, ArC NHAr), 180.6 (2C, C=S), 180.8 (C=S).

3-(4-chlorophenyl)-1,1-bis{2-[3-(4-chlorophenyl)thioureido]ethyl}thiourea (**3**)

Yield 91%. ^1^H NMR (300 MHz, DMSO-d_6_): δ 3.76–3.79 (m, 4H, CH_2_NH), 3.94–3.97 (m, 4H, NCH_2_), 7.35–7.43 (m, 12H, ArH), 7.99 (bs, 2H, NHCH_2_), 9.35 (s, 1H, NHAr), 9.78 (bs, 2H, NHAr); ^13^C NMR (75 MHz, DMSO-d_6_): δ 41.3 (2C, CH_2_), 49.5 (2C, CH_2_), 125.7 (4C, ArC *o*-NH), 127.8 (2C, ArC *o*-Cl), 127.9 (4C, ArC *o*-Cl), 128.5 (ArC *p*-NH), 128.6 (2C, ArC *p*-NH), 129.1 (2C, ArC *o*-NH), 137.8 (ArC *p*-Cl), 139.4 (2C, ArC *p*-Cl), 180.8 (2C, C=S), 180.9 (C=S).

3-(4-bromophenyl)-1,1-bis{2-[3-(4-bromophenyl)thioureido]ethyl}thiourea (**4**)

Yield 73%. ^1^H NMR (300 MHz, DMSO-d_6_): δ 3.74–3.79 (m, 4H, CH_2_NH), 3.94–3.98 (m, 4H, NCH_2_), 7.31–7.38 (m, 6H, ArH), 7.48–7.51 (m, 6H, ArH), 8.01 (bs, 2H, NHCH_2_), 9.34 (s, 1H, NHAr), 9.78 (bs, 2H, NHAr), ^13^C NMR (75 MHz, DMSO-d_6_): δ 44.2 (4C, CH_2_), 55.1 (2C, CH_2_), 126.3 (4C, ArC *o*-NH), 128.7 (2C, ArC *o*-Br), 129.1 (4C, ArC *o*-Br), 129.9 (ArC *p*-NH), 130.0 (2C, ArC *p*-NH), 132.3 (2C, ArC *o*-NH), 142.4 (ArC *p*-Br), 142.9 (2C, ArC *p*-Br), 181.1 (C=S), 181.6 (2C, C=S).

3-(3-chloro-4-fluorophenyl)-1,1-bis{2-[3-(3-chloro-4-fluorophenyl)thioureido]ethyl}thiourea (**5**)

Yield 81%. ^1^H NMR (300 MHz, DMSO-d_6_): δ 3.75–3.79 (m, 4H, CH_2_NH), 3.95–3.98 (m, 4H, NCH_2_), 7.27–7.39 (m, 6H, ArH), 7.56-7.59 (m, 1H, ArH *o*-F), 7.66–7.69 (m, 2H, ArH *o*-F), 8.03 (bs, 2H, NHCH_2_), 9.32 (s, 1H, NHAr), 9.78 (bs, 2H, NHAr), ^13^C NMR (75 MHz, DMSO-d_6_): δ 41.3 (2C, CH_2_), 49.5 (2C, CH_2_), 116.0 (d, ^2^*J* = 21.7 Hz, 2C), 116.7 (d, ^2^*J* = 21.9 Hz), 118.3 (d, ^2^*J* = 18.5 Hz, 2C), 119.0 (d, ^2^*J* = 18.0 Hz), 124.5 (ArC *m*-F), 125.8 (2C, ArC *m*-F), 126.8 (ArC *m*-F), 128.3 (2C, ArC *m*-F), 136.1 (ArC *p*-F), 137.7 (d, ^4^*J* = 3.3 Hz, 2C), 154.3 (d, ^1^*J* = 242.7 Hz, 2C), 154.6 (d, ^1^*J* = 243.4 Hz), 181.0 (3C, C=S).

3-phenethyl-1,1-bis[2-(3-phenethylthioureido)ethyl]thiourea (**6**)

Yield 76%. ^1^H NMR (300 MHz, DMSO-d6): δ 3.76–3.80 (m, 8H, CH_2_NH), 3.83–3.88 (m, 8H, CH_2_-Ar), 3.91–3.99 (m, 4H, NCH_2_), 7.22–7.39 (m, 15H, ArH), 7.89 (bs, 4H, NHCH2), 9.15 (s, 1H, NHCSN); 13C NMR (75 MHz, DMSO-d6): δ 36.2 (3C, CH_2_), 40.2 (3C, CH_2_), 48.7 (4C, CH_2_), 124.2 (ArC), 124.9 (2C, ArC), 125.7 (3C, ArC), 126.2 (4C, ArC), 127.1 (2C, ArC), 128.3 (ArC), 129.0 (2C, ArC), 139.1 (ArC), 141.7 (2C, ArC), 180.2 (2C, C=S), 180.6 (C=S).

3-(4-methoxyphenyl)-1,1-bis(2-{[1-(4-methoxyphenyl)-1H-tetrazol-5-yl]amino}ethyl)thiourea (**7**)

Triethylamine (503 μm, 3.75 mmol, 1–3 drops) was added to a suspension of **1** (1.25 mmol), sodium azide (244 mg, 3.75 mmol), and mercuric chloride (373 mg, 1.38 mmol) in 20 mL of dry DMF. The resulting suspension was stirred for 6 h at room temperature or until TLC indicated complete consumption of starting material. The suspension was filtered through a pad of celite, washing with CH_2_Cl_2_. The filtrate was diluted with water, and extracted with 3 × 15 mL of CH_2_Cl_2_. The combined organics were dried over MgSO_4_, filtered, and concentrated under reduced pressure. The resulting residue was purified by silica gel chromatography. Yield 83%. ^1^H NMR (300 MHz, DMSO-d_6_): δ 3.55–3.59 (m, 4H, CH_2_NH), 3.76 (s, 3H, OCH_3_), 3.83 (s, 6H, OCH_3_), 3.93–3.98 (m, 2H, NCH_2_), 6.87–6.90 (m, 2H, ArH *o*-NH), 7.05 (t, *J* = 5.1 Hz, 2H, NHCH_2_), 7.11–7.16 (m, 4H, ArH *o*-OCH_3_), 7.17–7.20 (m, 2H, ArH *o*-OCH_3_), 7.45–7.50 (m, 4H, ArH *o*-NH), 9.29 (s, 1H, NHAr), ^13^C NMR (75 MHz, DMSO-d_6_): δ 41.4 (2C, CH_2_), 49.5 (2C, CH_2_), 55.2 (OCH_3_), 55.6 (2C, OCH_3_), 113.1 (2C, ArC *o*-OCH_3_), 114.9 (4C, ArC *o*-OCH_3_), 125.6 (2C, ArC *o*-NH), 126.6 (4C, ArC *o*-N), 127.9 (2C, ArC *p*-OCH_3_), 133.6 (ArC *p*-OCH_3_), 155.1 (C=N), 156.7 (ArC *p*-NH), 159.9 (2C, ArC *p*-N), 181.3 (C=S).

### 4.3. Biological Assays

Description related to conducted biological studies including cell culture, suitable conditions, and methodology was presented in our previous paper [34].

## Figures and Tables

**Table 1:** Lipophilicity calculation and drug likeness for synthesized compounds.

Compound	MW [g/mol]	Log Po/w *	Drug Likeness **
**1**	598.80	2.17	Yes; 1 violation: MW > 500
**2**	508.73	3.13	Yes; 1 violation: MW > 500
**3**	612.06	4.54	No; 2 violations: MW > 500, MLOGP > 4.15
**4**	745.41	4.83	No; 2 violations: MW > 500, MLOGP > 4.15
**5**	666.03	5.64	No; 2 violations: MW > 500, MLOGP > 4.15
**6**	592.88	3.51	Yes; 1 violation: MW > 500
**7**	598.66	3.47	No; 2 violations: MW > 500, NorO > 10

* MLOGP, ** Lipinski rule.

**Table 2:** Crystal data and structure refinement for 3-(4-methoxyphenyl)-1,1-bis{2-[3-(4-methoxyphenyl)thioureido]ethyl}thiourea (**1**) and 3-(4-methoxyphenyl)-1,1-bis(2-{[1-(4-methoxyphenyl)-1H-tetrazol-5-yl]amino}ethyl)thiourea (**7**).

Compound	(1)	(7)
Empirical formula	C_28_H_34_N_6_O_3_S_3_	C_28_H_32_N_12_O_3_S_1_
Formula weight	598.79	616.72
Temperature [K]	100	130
Space group	P-1	P-1
Unit cell dimensions		
*a* [Å]	8.9243(5)	8.6567(8)
*b* [Å]	10.5616(5)	10.0605(8)
*c* [Å]	15.9877(8)	17.3294(13)
α [°]	83.818(2)	93.374(4)
β [°]	74.557(2)	95.721(5)
γ [°]	82.385(2)	106.197(4)
Volume *V* [Å^3^]	1435.56(13)	1436.2(2)
Z [molecules/cell]	2	2
*D*_calculated_ [g cm^−3^]	1.385	1.426
Absorption coefficientμ/mm^−1^	0.300	0.168
*θ* range for data collection [°]	3.008-24.999	2.117-24.999
Limiting indices	−10 < = *h* = > 10	−10 < = *h* = > 10
	−12 < = *k* = > 12	−11 < = *k* = > 11
	−19 < = *l* = > 19	−20 < = *l* = > 20
Reflections collected/unique	41,435/5062	39,822/5009
Data/parameters	5062/384	5009/412
Goodness of Fit	1.054	1.096
Final *R* index (*I* > 2σ)	0.0266	0.0560
w*R*^2^	0.0644	0.1310
Largest diff. Peak and hole [Å^−3^]	0.23 and −0.25	0.53 and −0.3

**Table 3:** Cytotoxic activity of tested compounds estimated by the MTT assay.

Compound	Cancer Cells						Normal Cells
	CaCo-2 *^c^*		A549 *^d^*		HTB-140 *^e^*		HaCaT *^f^*
	IC_50_ *^a^*	SI ^b^	IC_50_	SI	IC_50_	SI	IC_50_
**1**	>100	-	>100	-	>100	-	>100
**2**	>100	-	>100	-	>100	-	>100
**3**	27.70 ± 3.7	1.08	25.00 ± 2.4	1.19	22.63 ± 2.0	1.32	29.90 ± 1.8
**4**	17.15 ±1.9	1.12	16.79 ± 1.9	1.15	14.74 ± 1.5	1.35	19.30 ± 3.5
**5**	17.87 ± 2.8	1.05	15.43 ± 2.1	1.21	14.77 ± 3.2	1.27	18.75 ± 4.3
**6**	15.74 ± 1.7	1.02	14.98 ± 1.7	1.08	13.95 ± 2.5	1.16	16.15 ± 5.7
**7**	>100	-	>100	-	>100	-	>100
**Cisplatin**	1.68 ± 0.94	1.69	1.95 ± 0.83	1.46	1.13 ± 0.19	2.51	2.84 ± 1.06
**Doxorubicin**	0.53 ± 0.23	2.06	0.63 ± 0.21	1.73	0.47 ± 0.18	2.32	1.09 ± 0.23

*^a^* IC_50_ value—the concentration of a compound that corresponds to a 50% growth inhibition. Data are expressed as mean ± SD. *^b^* The SI (Selectivity Index) was calculated as: SI = IC_50_ for normal cell line/IC_50_ cancer cell line. *^c^* Human colorectal adenocarcinoma cell line (CaCo-2), *^d^* Human epithelial lung carcinoma cell line (A549), *^e^* Human melanoma cell line (HTB-140) and *^f^* Human immortal keratinocyte cell line from adult human skin (HaCaT).

## Supplementary Materials

The following are available online. Figure S1: The effect of compounds **3**-**6** on early and late apoptosis or necrosis in HaCaT HTB-140 and CaCo-2 cells detected with Annexin V-FITC/ 7-AAD by flow cytometry The lower right quadrant shows early apoptotic cells Annexin V-FITC positive and 7-AAD negative staining The upper right and upper left quadrants represent late stage of apoptotic or necrotic cells Annexin V-FITC positive 7-AAD positive Annexin V-FITC negative and 7-AAD positive staining respectively The lower left quadrant represents unstained A549 cells (Annexin V-FITC negative, 7-AAD negative).
