# Supplementary material for: Cytotoxicity Evaluation of Novel bis(2-aminoethyl)amine Derivatives
Source: Molecules. 2020 Jun 18;25(12):2816. doi: 10.3390/molecules25122816 (PMC7355942; doi:10.3390/molecules25122816)
Supplement: Supplementary file 1 [file molecules-25-02816-s001.pdf]

HaCaT

Control

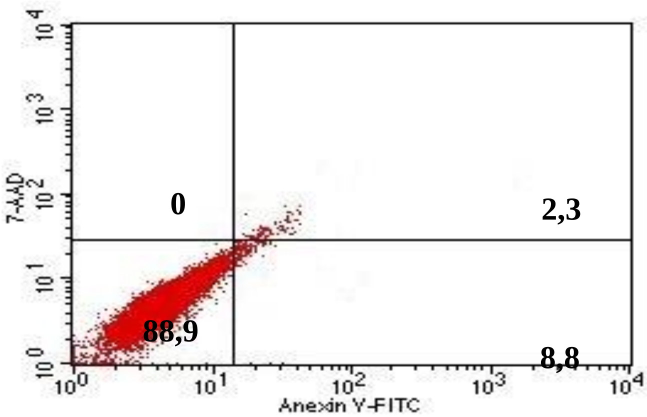

3

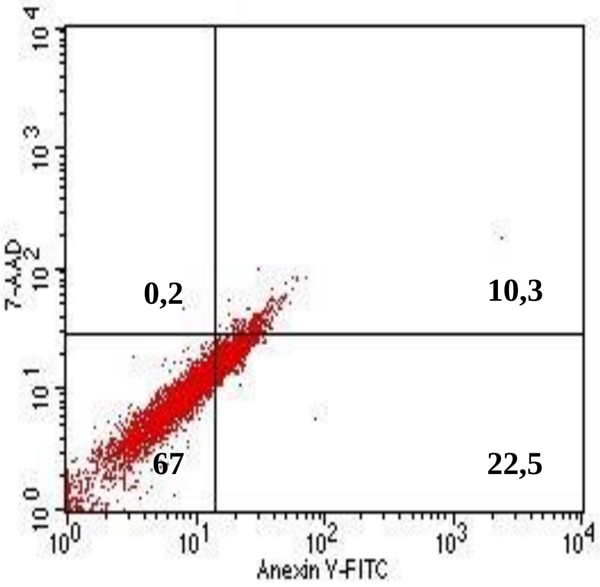

4

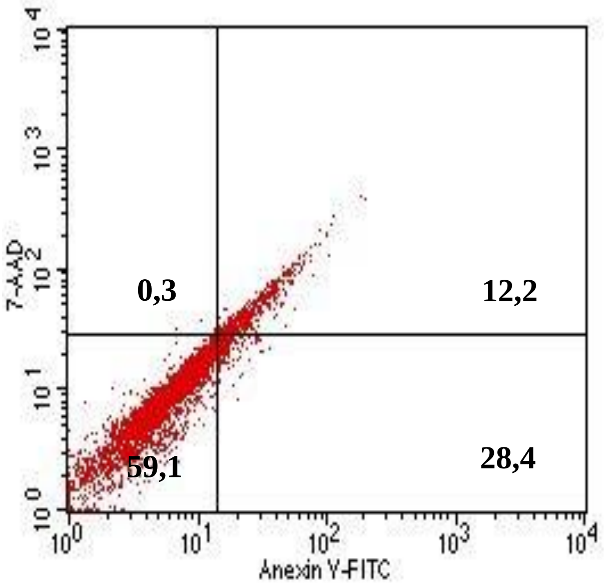

5

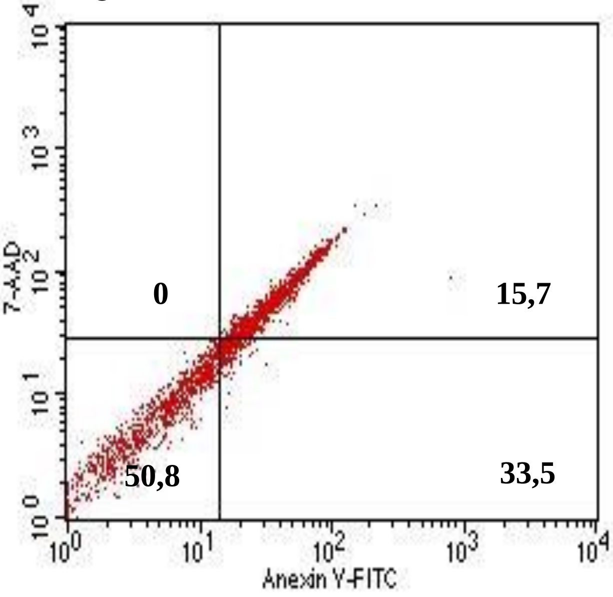

6

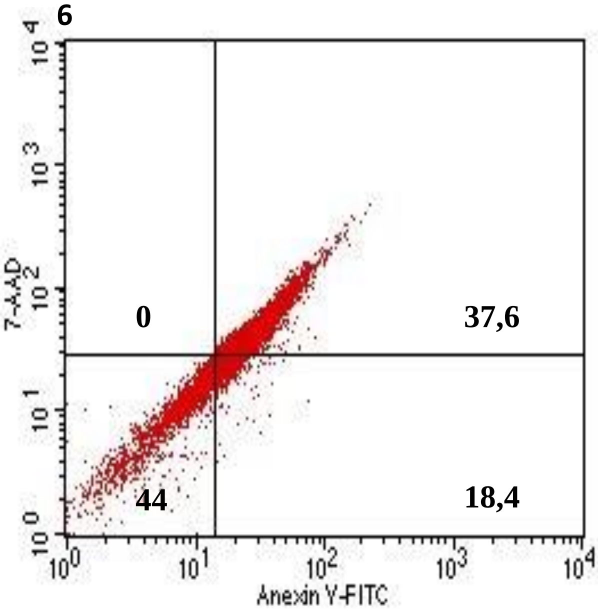

CaCo-2

Control

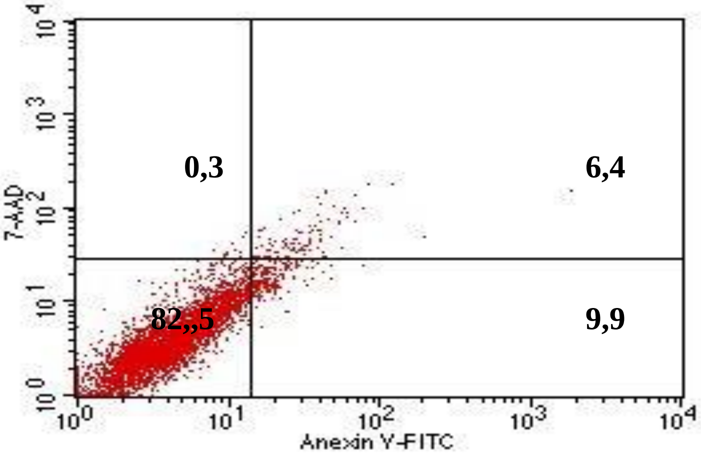

3

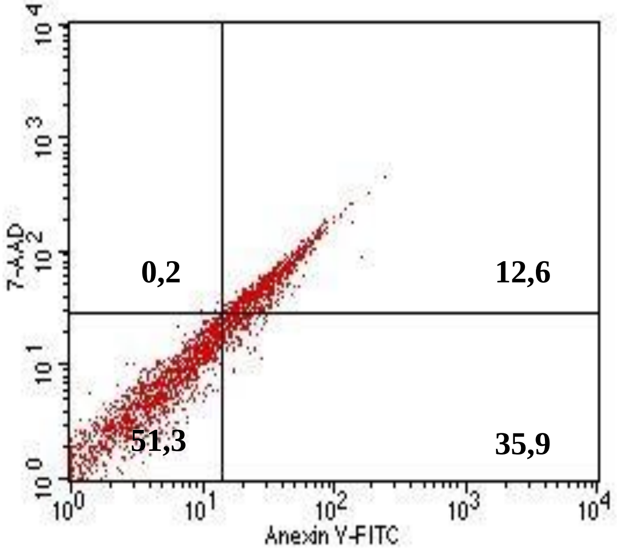

4

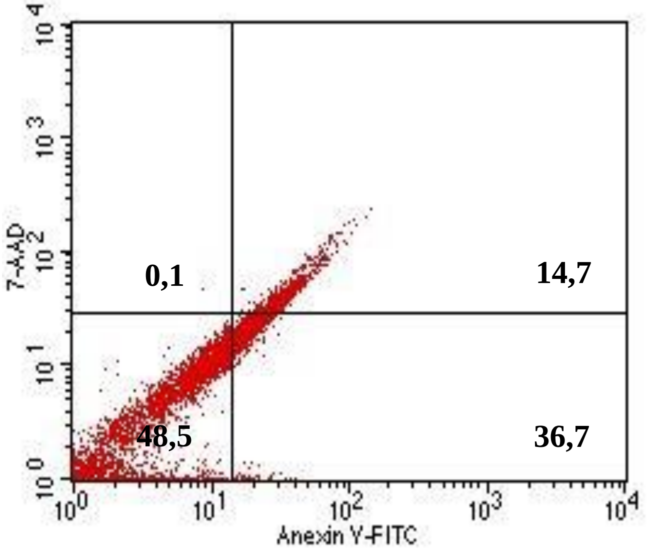

5

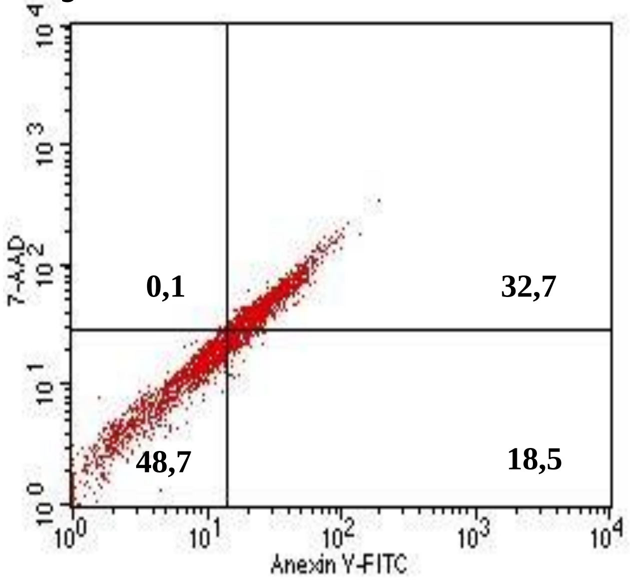

6

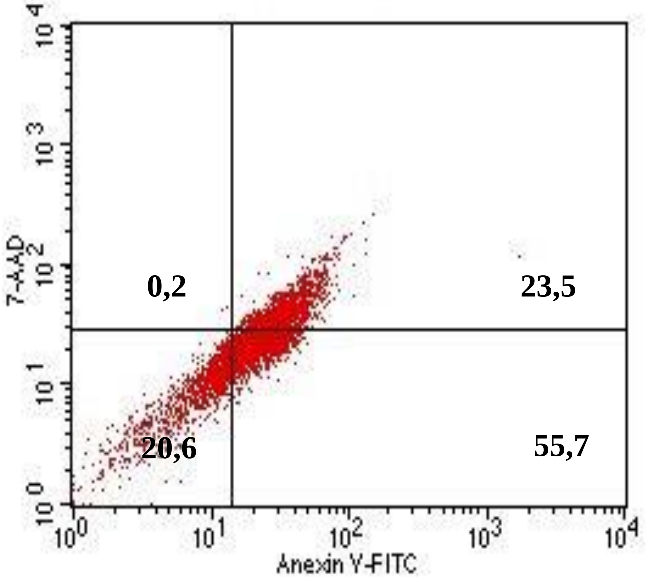

HTB-140 **Control**

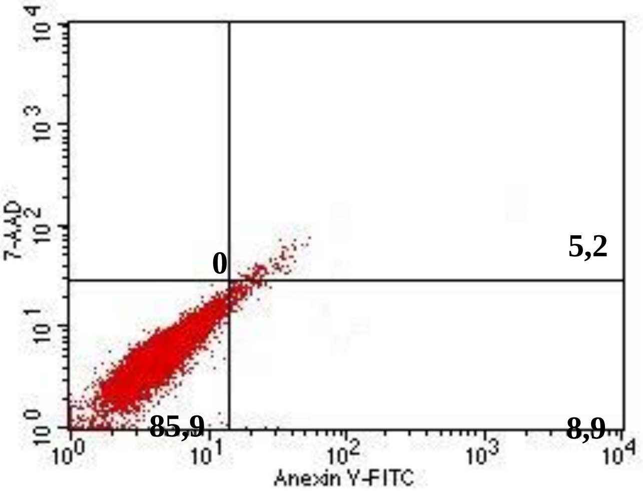

3

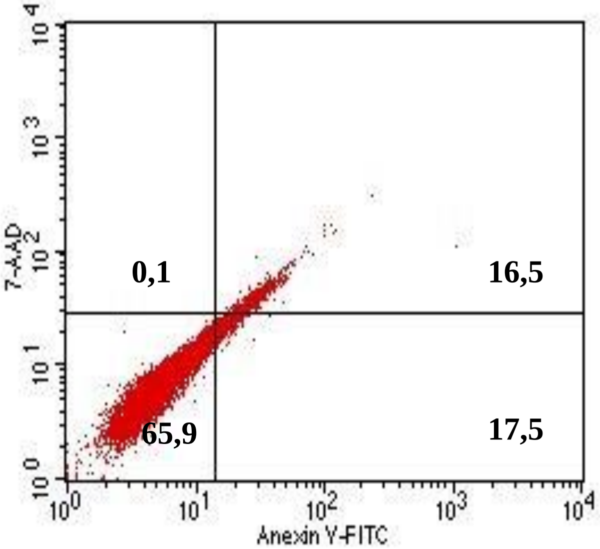

4

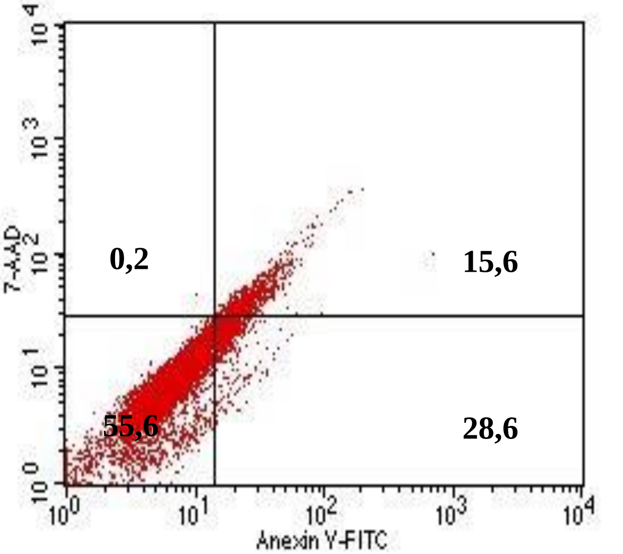

5

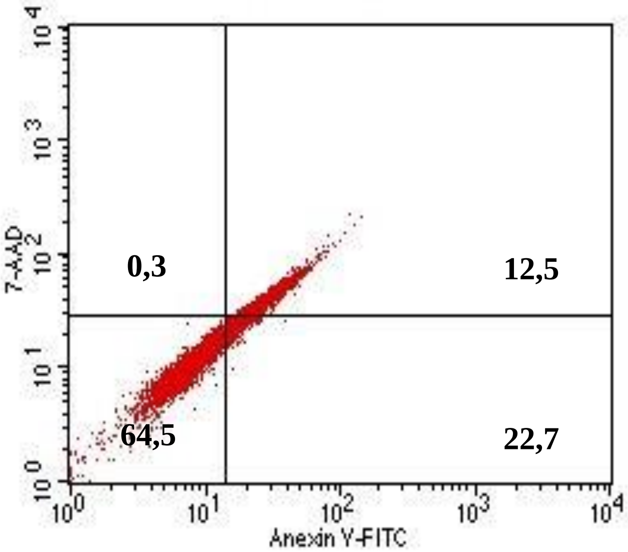

6

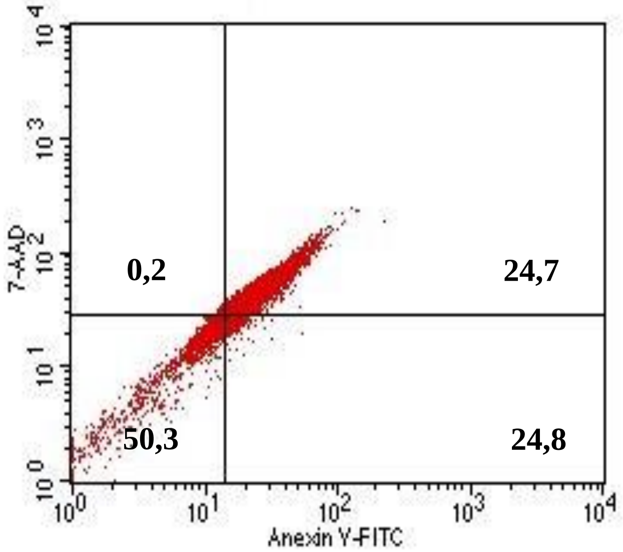

Fig. 1S. The effect of compounds **3-6** on early and late apoptosis or necrosis in HaCaT, HTB-140 and CaCo-2 cells, detected with Annexin V-FITC/7-AAD by flow cytometry. The lower right quadrant shows early apoptotic cells (Annexin V-FITC positive and 7-AAD negative staining). The upper right and upper left quadrants represent late stage of apoptotic or necrotic cells (Annexin V-FITC positive, 7-AAD positive, Annexin V-FITC negative and 7-AAD positive staining, respectively). The lower left quadrant represents unstained A549 cells (Annexin V-FITC negative, 7-AAD negative).
